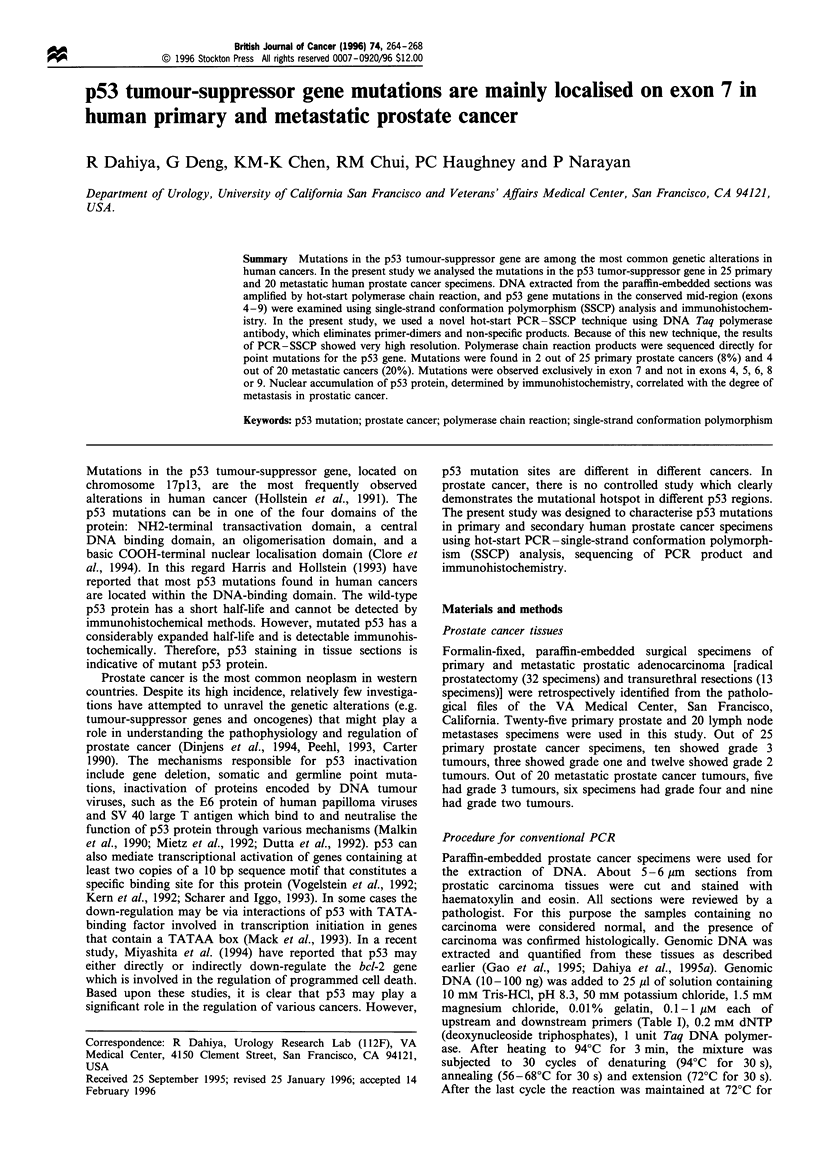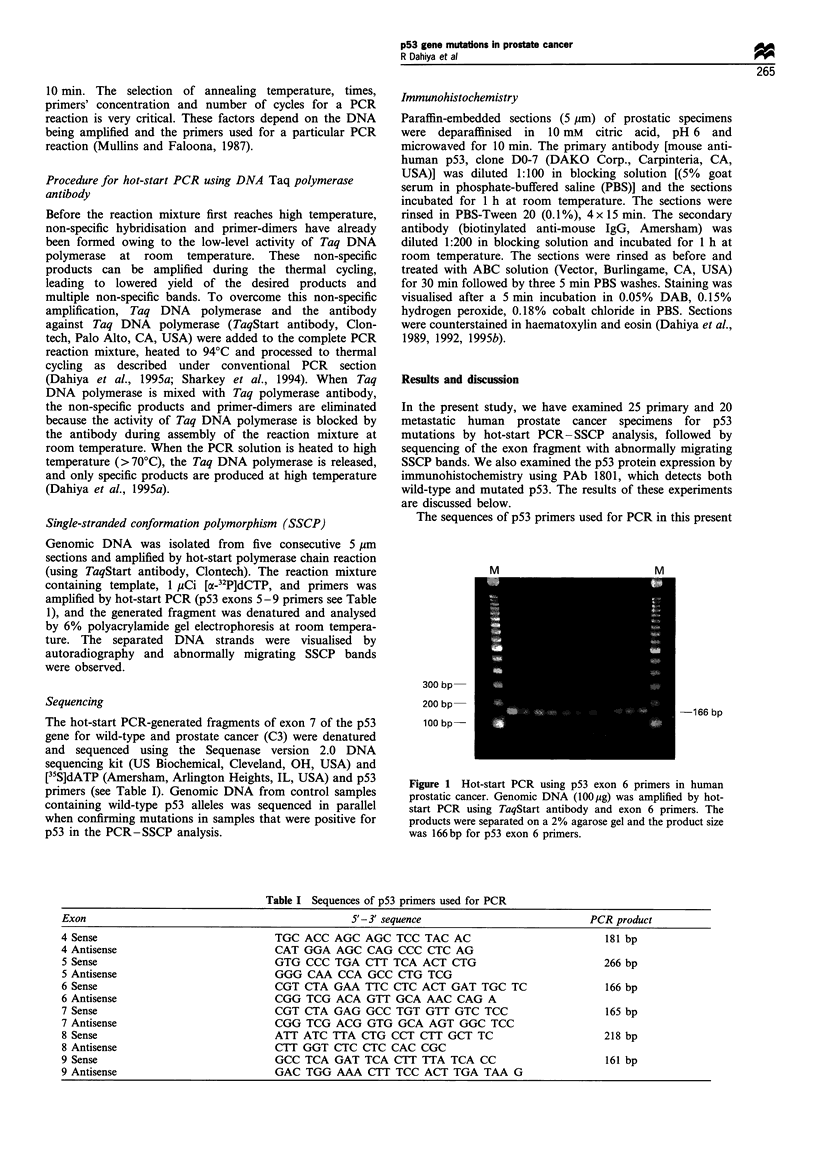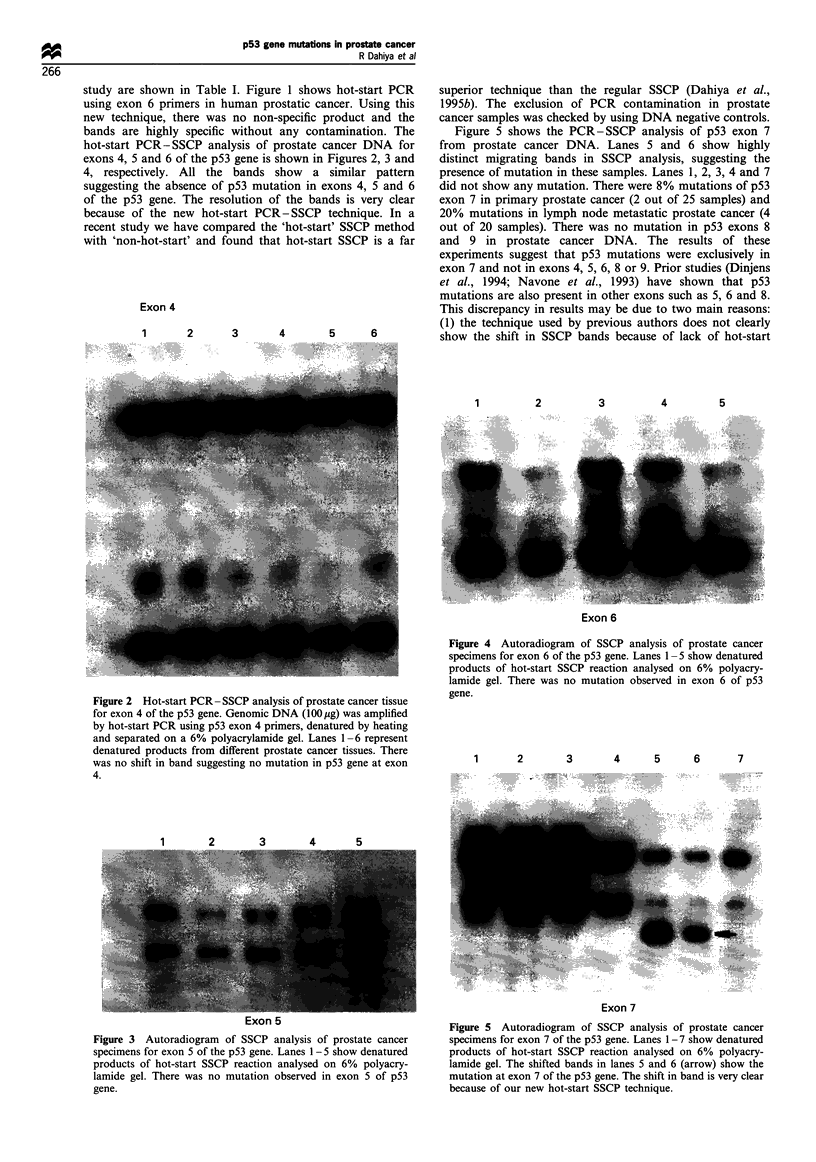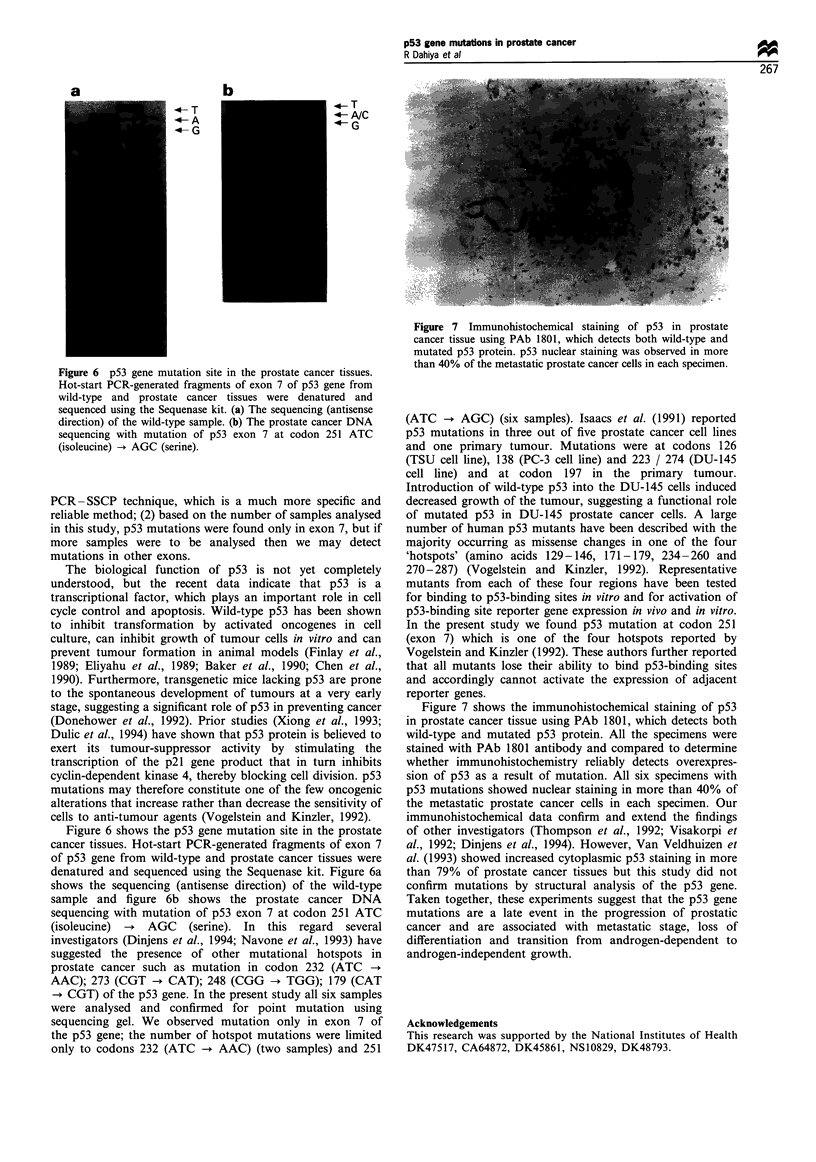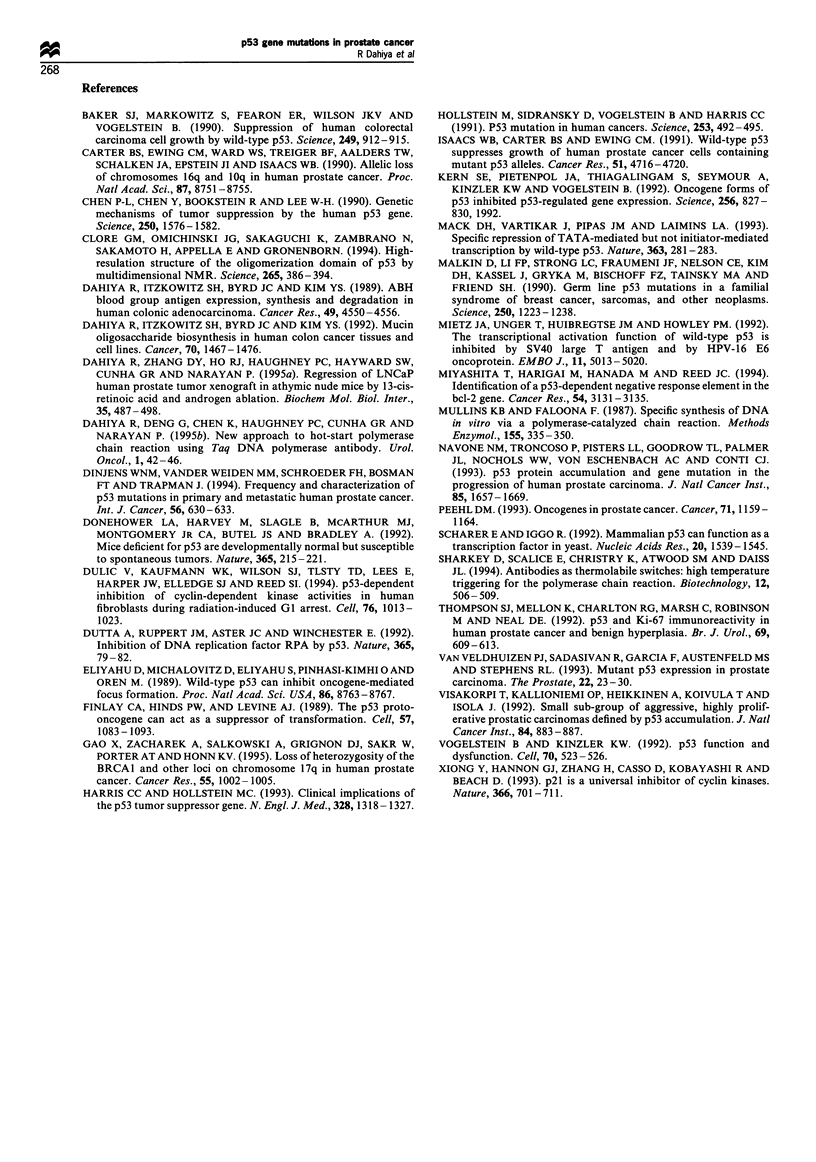# P53 tumour-suppressor gene mutations are mainly localised on exon 7 in human primary and metastatic prostate cancer.

**DOI:** 10.1038/bjc.1996.349

**Published:** 1996-07

**Authors:** R. Dahiya, G. Deng, K. M. Chen, R. M. Chui, P. C. Haughney, P. Narayan

**Affiliations:** Department of Urology, University of California San Francisco, USA.

## Abstract

**Images:**


British Journal of Cancer (1996) 74, 264-268
© 1996 Stockton Press All rights reserved 0007-0920/96 $12.00

### p53 tumour-suppressor gene mutations are mainly localised on exon 7 in human primary and metastatic prostate cancer

R Dahiya, G Deng, KM-K Chen, RM Chui, PC Haughney and P Narayan

*Department of Urology, University of California San Francisco and Veterans' Affairs Medical Center, San Francisco, CA 94121, USA.*

**Summary** Mutations in the p53 tumour-suppressor gene are among the most common genetic alterations in human cancers. In the present study we analysed the mutations in the p53 tumor-suppressor gene in 25 primary and 20 metastatic human prostate cancer specimens. DNA extracted from the paraffin-embedded sections was amplified by hot-start polymerase chain reaction, and p53 gene mutations in the conserved mid-region (exons 4-9) were examined using single-strand conformation polymorphism (SSCP) analysis and immunohistochemistry. In the present study, we used a novel hot-start PCR-SSCP technique using DNA *Taq* polymerase antibody, which eliminates primer-dimers and non-specific products. Because of this new technique, the results of PCR-SSCP showed very high resolution. Polymerase chain reaction products were sequenced directly for point mutations for the p53 gene. Mutations were found in 2 out of 25 primary prostate cancers (8%) and 4 out of 20 metastatic cancers (20%). Mutations were observed exclusively in exon 7 and not in exons 4, 5, 6, 8 or 9. Nuclear accumulation of p53 protein, determined by immunohistochemistry, correlated with the degree of metastasis in prostatic cancer.

**Keywords:** p53 mutation; prostate cancer; polymerase chain reaction; single-strand conformation polymorphism

Mutations in the p53 tumour-suppressor gene, located on chromosome 17p13, are the most frequently observed alterations in human cancer (Hollstein *et al.*, 1991). The p53 mutations can be in one of the four domains of the protein: NH2-terminal transactivation domain, a central DNA binding domain, an oligomerisation domain, and a basic COOH-terminal nuclear localisation domain (Clore *et al.*, 1994). In this regard Harris and Hollstein (1993) have reported that most p53 mutations found in human cancers are located within the DNA-binding domain. The wild-type p53 protein has a short half-life and cannot be detected by immunohistochemical methods. However, mutated p53 has a considerably expanded half-life and is detectable immunohistochemically. Therefore, p53 staining in tissue sections is indicative of mutant p53 protein.

Prostate cancer is the most common neoplasm in western countries. Despite its high incidence, relatively few investigations have attempted to unravel the genetic alterations (e.g. tumour-suppressor genes and oncogenes) that might play a role in understanding the pathophysiology and regulation of prostate cancer (Dinjens *et al.*, 1994, Peehl, 1993, Carter 1990). The mechanisms responsible for p53 inactivation include gene deletion, somatic and germline point mutations, inactivation of proteins encoded by DNA tumour viruses, such as the E6 protein of human papilloma viruses and SV 40 large T antigen which bind to and neutralise the function of p53 protein through various mechanisms (Malkin *et al.*, 1990; Mietz *et al.*, 1992; Dutta *et al.*, 1992). p53 can also mediate transcriptional activation of genes containing at least two copies of a 10 bp sequence motif that constitutes a specific binding site for this protein (Vogelstein *et al.*, 1992; Kern *et al.*, 1992; Scharer and Iggo, 1993). In some cases the down-regulation may be via interactions of p53 with TATA-binding factor involved in transcription initiation in genes that contain a TATAA box (Mack *et al.*, 1993). In a recent study, Miyashita *et al.* (1994) have reported that p53 may either directly or indirectly down-regulate the *bcl*-2 gene which is involved in the regulation of programmed cell death. Based upon these studies, it is clear that p53 may play a significant role in the regulation of various cancers. However, p53 mutation sites are different in different cancers. In prostate cancer, there is no controlled study which clearly demonstrates the mutational hotspot in different p53 regions. The present study was designed to characterise p53 mutations in primary and secondary human prostate cancer specimens using hot-start PCR-single-strand conformation polymorphism (SSCP) analysis, sequencing of PCR product and immunohistochemistry.

Correspondence: R Dahiya, Urology Research Lab (112F), VA Medical Center, 4150 Clement Street, San Francisco, CA 94121, USA
Received 25 September 1995; revised 25 January 1996; accepted 14 February 1996

### Materials and methods

### *Prostate cancer tissues*

Formalin-fixed, paraffin-embedded surgical specimens of primary and metastatic prostatic adenocarcinoma [radical prostatectomy (32 specimens) and transurethral resections (13 specimens)] were retrospectively identified from the pathological files of the VA Medical Center, San Francisco, California. Twenty-five primary prostate and 20 lymph node metastases specimens were used in this study. Out of 25 primary prostate cancer specimens, ten showed grade 3 tumours, three showed grade one and twelve showed grade 2 tumours. Out of 20 metastatic prostate cancer tumours, five had grade 3 tumours, six specimens had grade four and nine had grade two tumours.

### *Procedure for conventional PCR*

Paraffin-embedded prostate cancer specimens were used for the extraction of DNA. About 5-6 $\mu$m sections from prostatic carcinoma tissues were cut and stained with haematoxylin and eosin. All sections were reviewed by a pathologist. For this purpose the samples containing no carcinoma were considered normal, and the presence of carcinoma was confirmed histologically. Genomic DNA was extracted and quantified from these tissues as described earlier (Gao *et al.*, 1995; Dahiya *et al.*, 1995*a*). Genomic DNA (10-100 ng) was added to 25 $\mu$l of solution containing 10 mM Tris-HCl, pH 8.3, 50 mM potassium chloride, 1.5 mM magnesium chloride, 0.01% gelatin, 0.1-1 $\mu$M each of upstream and downstream primers (Table I), 0.2 mM dNTP (deoxynucleoside triphosphates), 1 unit *Taq* DNA polymerase. After heating to 94°C for 3 min, the mixture was subjected to 30 cycles of denaturing (94°C for 30 s), annealing (56-68°C for 30 s) and extension (72°C for 30 s). After the last cycle the reaction was maintained at 72°C for

10 min. The selection of annealing temperature, times, primers' concentration and number of cycles for a PCR reaction is very critical. These factors depend on the DNA being amplified and the primers used for a particular PCR reaction (Mullins and Faloona, 1987).

*Procedure for hot-start PCR using DNA* Taq *polymerase antibody*

Before the reaction mixture first reaches high temperature, non-specific hybridisation and primer-dimers have already been formed owing to the low-level activity of *Taq* DNA polymerase at room temperature. These non-specific products can be amplified during the thermal cycling, leading to lowered yield of the desired products and multiple non-specific bands. To overcome this non-specific amplification, *Taq* DNA polymerase and the antibody against *Taq* DNA polymerase (*Taq*Start antibody, Clontech, Palo Alto, CA, USA) were added to the complete PCR reaction mixture, heated to 94°C and processed to thermal cycling as described under conventional PCR section (Dahiya *et al.*, 1995*a*; Sharkey *et al.*, 1994). When *Taq* DNA polymerase is mixed with *Taq* polymerase antibody, the non-specific products and primer-dimers are eliminated because the activity of *Taq* DNA polymerase is blocked by the antibody during assembly of the reaction mixture at room temperature. When the PCR solution is heated to high temperature (>70°C), the *Taq* DNA polymerase is released, and only specific products are produced at high temperature (Dahiya *et al.*, 1995*a*).

*Single-stranded conformation polymorphism (SSCP)*

Genomic DNA was isolated from five consecutive 5 μm sections and amplified by hot-start polymerase chain reaction (using *Taq*Start antibody, Clontech). The reaction mixture containing template, 1 μCi [α-$^{32}$P]dCTP, and primers was amplified by hot-start PCR (p53 exons 5–9 primers see Table 1), and the generated fragment was denatured and analysed by 6% polyacrylamide gel electrophoresis at room temperature. The separated DNA strands were visualised by autoradiography and abnormally migrating SSCP bands were observed.

*Sequencing*

The hot-start PCR-generated fragments of exon 7 of the p53 gene for wild-type and prostate cancer (C3) were denatured and sequenced using the Sequenase version 2.0 DNA sequencing kit (US Biochemical, Cleveland, OH, USA) and [$^{35}$S]dATP (Amersham, Arlington Heights, IL, USA) and p53 primers (see Table I). Genomic DNA from control samples containing wild-type p53 alleles was sequenced in parallel when confirming mutations in samples that were positive for p53 in the PCR–SSCP analysis.

*Immunohistochemistry*

Paraffin-embedded sections (5 μm) of prostatic specimens were deparaffinised in 10 mM citric acid, pH 6 and microwaved for 10 min. The primary antibody [mouse anti-human p53, clone D0-7 (DAKO Corp., Carpinteria, CA, USA)] was diluted 1:100 in blocking solution [(5% goat serum in phosphate-buffered saline (PBS)] and the sections incubated for 1 h at room temperature. The sections were rinsed in PBS-Tween 20 (0.1%), 4 × 15 min. The secondary antibody (biotinylated anti-mouse IgG, Amersham) was diluted 1:200 in blocking solution and incubated for 1 h at room temperature. The sections were rinsed as before and treated with ABC solution (Vector, Burlingame, CA, USA) for 30 min followed by three 5 min PBS washes. Staining was visualised after a 5 min incubation in 0.05% DAB, 0.15% hydrogen peroxide, 0.18% cobalt chloride in PBS. Sections were counterstained in haematoxylin and eosin (Dahiya *et al.*, 1989, 1992, 1995*b*).

### Results and discussion

In the present study, we have examined 25 primary and 20 metastatic human prostate cancer specimens for p53 mutations by hot-start PCR–SSCP analysis, followed by sequencing of the exon fragment with abnormally migrating SSCP bands. We also examined the p53 protein expression by immunohistochemistry using PAb 1801, which detects both wild-type and mutated p53. The results of these experiments are discussed below.

The sequences of p53 primers used for PCR in this present

**Figure 1** Hot-start PCR using p53 exon 6 primers in human prostatic cancer. Genomic DNA (100 μg) was amplified by hot-start PCR using *Taq*Start antibody and exon 6 primers. The products were separated on a 2% agarose gel and the product size was 166 bp for p53 exon 6 primers.

**Table I** Sequences of p53 primers used for PCR

| *Exon* | *5′–3′ sequence* | *PCR product* |
|---|---|---|
| 4 Sense | TGC ACC AGC AGC TCC TAC AC | 181 bp |
| 4 Antisense | CAT GGA AGC CAG CCC CTC AG | |
| 5 Sense | GTG CCC TGA CTT TCA ACT CTG | 266 bp |
| 5 Antisense | GGG CAA CCA GCC CTG TCG | |
| 6 Sense | CGT CTA GAA TTC CTC ACT GAT TGC TC | 166 bp |
| 6 Antisense | CGG TCG ACA GTT GCA AAC CAG A | |
| 7 Sense | CGT CTA GAG GCC TGT GTT GTC TCC | 165 bp |
| 7 Antisense | CGG TCG ACG GTG GCA AGT GGC TCC | |
| 8 Sense | ATT ATC TTA CTG CCT CTT GCT TC | 218 bp |
| 8 Antisense | CTT GGT CTC CTC CAC CGC | |
| 9 Sense | GCC TCA GAT TCA CTT TTA TCA CC | 161 bp |
| 9 Antisense | GAC TGG AAA CTT TCC ACT TGA TAA G | |

study are shown in Table I. Figure 1 shows hot-start PCR using exon 6 primers in human prostatic cancer. Using this new technique, there was no non-specific product and the bands are highly specific without any contamination. The hot-start PCR–SSCP analysis of prostate cancer DNA for exons 4, 5 and 6 of the p53 gene is shown in Figures 2, 3 and 4, respectively. All the bands show a similar pattern suggesting the absence of p53 mutation in exons 4, 5 and 6 of the p53 gene. The resolution of the bands is very clear because of the new hot-start PCR–SSCP technique. In a recent study we have compared the 'hot-start' SSCP method with 'non-hot-start' and found that hot-start SSCP is a far superior technique than the regular SSCP (Dahiya et al., 1995b). The exclusion of PCR contamination in prostate cancer samples was checked by using DNA negative controls.

Figure 5 shows the PCR–SSCP analysis of p53 exon 7 from prostate cancer DNA. Lanes 5 and 6 show highly distinct migrating bands in SSCP analysis, suggesting the presence of mutation in these samples. Lanes 1, 2, 3, 4 and 7 did not show any mutation. There were 8% mutations of p53 exon 7 in primary prostate cancer (2 out of 25 samples) and 20% mutations in lymph node metastatic prostate cancer (4 out of 20 samples). There was no mutation in p53 exons 8 and 9 in prostate cancer DNA. The results of these experiments suggest that p53 mutations were exclusively in exon 7 and not in exons 4, 5, 6, 8 or 9. Prior studies (Dinjens et al., 1994; Navone et al., 1993) have shown that p53 mutations are also present in other exons such as 5, 6 and 8. This discrepancy in results may be due to two main reasons: (1) the technique used by previous authors does not clearly show the shift in SSCP bands because of lack of hot-start

**Figure 2** Hot-start PCR–SSCP analysis of prostate cancer tissue for exon 4 of the p53 gene. Genomic DNA (100 μg) was amplified by hot-start PCR using p53 exon 4 primers, denatured by heating and separated on a 6% polyacrylamide gel. Lanes 1–6 represent denatured products from different prostate cancer tissues. There was no shift in band suggesting no mutation in p53 gene at exon 4.

**Figure 3** Autoradiogram of SSCP analysis of prostate cancer specimens for exon 5 of the p53 gene. Lanes 1–5 show denatured products of hot-start SSCP reaction analysed on 6% polyacrylamide gel. There was no mutation observed in exon 5 of p53 gene.

**Figure 4** Autoradiogram of SSCP analysis of prostate cancer specimens for exon 6 of the p53 gene. Lanes 1–5 show denatured products of hot-start SSCP reaction analysed on 6% polyacrylamide gel. There was no mutation observed in exon 6 of p53 gene.

**Figure 5** Autoradiogram of SSCP analysis of prostate cancer specimens for exon 7 of the p53 gene. Lanes 1–7 show denatured products of hot-start SSCP reaction analysed on 6% polyacrylamide gel. The shifted bands in lanes 5 and 6 (arrow) show the mutation at exon 7 of the p53 gene. The shift in band is very clear because of our new hot-start SSCP technique.

**Figure 6** p53 gene mutation site in the prostate cancer tissues. Hot-start PCR-generated fragments of exon 7 of p53 gene from wild-type and prostate cancer tissues were denatured and sequenced using the Sequenase kit. (**a**) The sequencing (antisense direction) of the wild-type sample. (**b**) The prostate cancer DNA sequencing with mutation of p53 exon 7 at codon 251 ATC (isoleucine) → AGC (serine).

PCR–SSCP technique, which is a much more specific and reliable method; (2) based on the number of samples analysed in this study, p53 mutations were found only in exon 7, but if more samples were to be analysed then we may detect mutations in other exons.

The biological function of p53 is not yet completely understood, but the recent data indicate that p53 is a transcriptional factor, which plays an important role in cell cycle control and apoptosis. Wild-type p53 has been shown to inhibit transformation by activated oncogenes in cell culture, can inhibit growth of tumour cells *in vitro* and can prevent tumour formation in animal models (Finlay *et al.*, 1989; Eliyahu *et al.*, 1989; Baker *et al.*, 1990; Chen *et al.*, 1990). Furthermore, transgenetic mice lacking p53 are prone to the spontaneous development of tumours at a very early stage, suggesting a significant role of p53 in preventing cancer (Donehower *et al.*, 1992). Prior studies (Xiong *et al.*, 1993; Dulic *et al.*, 1994) have shown that p53 protein is believed to exert its tumour-suppressor activity by stimulating the transcription of the p21 gene product that in turn inhibits cyclin-dependent kinase 4, thereby blocking cell division. p53 mutations may therefore constitute one of the few oncogenic alterations that increase rather than decrease the sensitivity of cells to anti-tumour agents (Vogelstein and Kinzler, 1992).

Figure 6 shows the p53 gene mutation site in the prostate cancer tissues. Hot-start PCR-generated fragments of exon 7 of p53 gene from wild-type and prostate cancer tissues were denatured and sequenced using the Sequenase kit. Figure 6a shows the sequencing (antisense direction) of the wild-type sample and figure 6b shows the prostate cancer DNA sequencing with mutation of p53 exon 7 at codon 251 ATC (isoleucine) → AGC (serine). In this regard several investigators (Dinjens *et al.*, 1994; Navone *et al.*, 1993) have suggested the presence of other mutational hotspots in prostate cancer such as mutation in codon 232 (ATC → AAC); 273 (CGT → CAT); 248 (CGG → TGG); 179 (CAT → CGT) of the p53 gene. In the present study all six samples were analysed and confirmed for point mutation using sequencing gel. We observed mutation only in exon 7 of the p53 gene; the number of hotspot mutations were limited only to codons 232 (ATC → AAC) (two samples) and 251 (ATC → AGC) (six samples). Isaacs *et al.* (1991) reported p53 mutations in three out of five prostate cancer cell lines and one primary tumour. Mutations were at codons 126 (TSU cell line), 138 (PC-3 cell line) and 223 / 274 (DU-145 cell line) and at codon 197 in the primary tumour. Introduction of wild-type p53 into the DU-145 cells induced decreased growth of the tumour, suggesting a functional role of mutated p53 in DU-145 prostate cancer cells. A large number of human p53 mutants have been described with the majority occurring as missense changes in one of the four 'hotspots' (amino acids 129–146, 171–179, 234–260 and 270–287) (Vogelstein and Kinzler, 1992). Representative mutants from each of these four regions have been tested for binding to p53-binding sites *in vitro* and for activation of p53-binding site reporter gene expression *in vivo* and *in vitro*. In the present study we found p53 mutation at codon 251 (exon 7) which is one of the four hotspots reported by Vogelstein and Kinzler (1992). These authors further reported that all mutants lose their ability to bind p53-binding sites and accordingly cannot activate the expression of adjacent reporter genes.

**Figure 7** Immunohistochemical staining of p53 in prostate cancer tissue using PAb 1801, which detects both wild-type and mutated p53 protein. p53 nuclear staining was observed in more than 40% of the metastatic prostate cancer cells in each specimen.

Figure 7 shows the immunohistochemical staining of p53 in prostate cancer tissue using PAb 1801, which detects both wild-type and mutated p53 protein. All the specimens were stained with PAb 1801 antibody and compared to determine whether immunohistochemistry reliably detects overexpression of p53 as a result of mutation. All six specimens with p53 mutations showed nuclear staining in more than 40% of the metastatic prostate cancer cells in each specimen. Our immunohistochemical data confirm and extend the findings of other investigators (Thompson *et al.*, 1992; Visakorpi *et al.*, 1992; Dinjens *et al.*, 1994). However, Van Veldhuizen *et al.* (1993) showed increased cytoplasmic p53 staining in more than 79% of prostate cancer tissues but this study did not confirm mutations by structural analysis of the p53 gene. Taken together, these experiments suggest that the p53 gene mutations are a late event in the progression of prostatic cancer and are associated with metastatic stage, loss of differentiation and transition from androgen-dependent to androgen-independent growth.

**Acknowledgements**
This research was supported by the National Institutes of Health DK47517, CA64872, DK45861, NS10829, DK48793.

### References

BAKER SJ, MARKOWITZ S, FEARON ER, WILSON JKV AND VOGELSTEIN B. (1990). Suppression of human colorectal carcinoma cell growth by wild-type p53. *Science*, **249**, 912–915.

CARTER BS, EWING CM, WARD WS, TREIGER BF, AALDERS TW, SCHALKEN JA, EPSTEIN JI AND ISAACS WB. (1990). Allelic loss of chromosomes 16q and 10q in human prostate cancer. *Proc. Natl Acad. Sci.*, **87**, 8751–8755.

CHEN P-L, CHEN Y, BOOKSTEIN R AND LEE W-H. (1990). Genetic mechanisms of tumor suppression by the human p53 gene. *Science*, **250**, 1576–1582.

CLORE GM, OMICHINSKI JG, SAKAGUCHI K, ZAMBRANO N, SAKAMOTO H, APPELLA E AND GRONENBORN. (1994). High-resulation structure of the oligomerization domain of p53 by multidimensional NMR. *Science*, **265**, 386–394.

DAHIYA R, ITZKOWITZ SH, BYRD JC AND KIM YS. (1989). ABH blood group antigen expression, synthesis and degradation in human colonic adenocarcinoma. *Cancer Res.*, **49**, 4550–4556.

DAHIYA R, ITZKOWITZ SH, BYRD JC AND KIM YS. (1992). Mucin oligosaccharide biosynthesis in human colon cancer tissues and cell lines. *Cancer*, **70**, 1467–1476.

DAHIYA R, ZHANG DY, HO RJ, HAUGHNEY PC, HAYWARD SW, CUNHA GR AND NARAYAN P. (1995*a*). Regression of LNCaP human prostate tumor xenograft in athymic nude mice by 13-cis-retinoic acid and androgen ablation. *Biochem Mol. Biol. Inter.*, **35**, 487–498.

DAHIYA R, DENG G, CHEN K, HAUGHNEY PC, CUNHA GR AND NARAYAN P. (1995*b*). New approach to hot-start polymerase chain reaction using *Taq* DNA polymerase antibody. *Urol. Oncol.*, **1**, 42–46.

DINJENS WNM, VANDER WEIDEN MM, SCHROEDER FH, BOSMAN FT AND TRAPMAN J. (1994). Frequency and characterization of p53 mutations in primary and metastatic human prostate cancer. *Int. J. Cancer*, **56**, 630–633.

DONEHOWER LA, HARVEY M, SLAGLE B, MCARTHUR MJ, MONTGOMERY JR CA, BUTEL JS AND BRADLEY A. (1992). Mice deficient for p53 are developmentally normal but susceptible to spontaneous tumors. *Nature*, **365**, 215–221.

DULIC V, KAUFMANN WK, WILSON SJ, TLSTY TD, LEES E, HARPER JW, ELLEDGE SJ AND REED SI. (1994). p53-dependent inhibition of cyclin-dependent kinase activities in human fibroblasts during radiation-induced G1 arrest. *Cell*, **76**, 1013–1023.

DUTTA A, RUPPERT JM, ASTER JC AND WINCHESTER E. (1992). Inhibition of DNA replication factor RPA by p53. *Nature*, **365**, 79–82.

ELIYAHU D, MICHALOVITZ D, ELIYAHU S, PINHASI-KIMHI O AND OREN M. (1989). Wild-type p53 can inhibit oncogene-mediated focus formation. *Proc. Natl Acad. Sci. USA*, **86**, 8763–8767.

FINLAY CA, HINDS PW, AND LEVINE AJ. (1989). The p53 proto-oncogene can act as a suppressor of transformation. *Cell*, **57**, 1083–1093.

GAO X, ZACHAREK A, SALKOWSKI A, GRIGNON DJ, SAKR W, PORTER AT AND HONN KV. (1995). Loss of heterozygosity of the BRCA1 and other loci on chromosome 17q in human prostate cancer. *Cancer Res.*, **55**, 1002–1005.

HARRIS CC AND HOLLSTEIN MC. (1993). Clinical implications of the p53 tumor suppressor gene. *N. Engl. J. Med.*, **328**, 1318–1327.

HOLLSTEIN M, SIDRANSKY D, VOGELSTEIN B AND HARRIS CC (1991). P53 mutation in human cancers. *Science*, **253**, 492–495.

ISAACS WB, CARTER BS AND EWING CM. (1991). Wild-type p53 suppresses growth of human prostate cancer cells containing mutant p53 alleles. *Cancer Res.*, **51**, 4716–4720.

KERN SE, PIETENPOL JA, THIAGALINGAM S, SEYMOUR A, KINZLER KW AND VOGELSTEIN B. (1992). Oncogene forms of p53 inhibited p53-regulated gene expression. *Science*, **256**, 827–830, 1992.

MACK DH, VARTIKAR J, PIPAS JM AND LAIMINS LA. (1993). Specific repression of TATA-mediated but not initiator-mediated transcription by wild-type p53. *Nature*, **363**, 281–283.

MALKIN D, LI FP, STRONG LC, FRAUMENI JF, NELSON CE, KIM DH, KASSEL J, GRYKA M, BISCHOFF FZ, TAINSKY MA AND FRIEND SH. (1990). Germ line p53 mutations in a familial syndrome of breast cancer, sarcomas, and other neoplasms. *Science*, **250**, 1223–1238.

MIETZ JA, UNGER T, HUIBREGTSE JM AND HOWLEY PM. (1992). The transcriptional activation function of wild-type p53 is inhibited by SV40 large T antigen and by HPV-16 E6 oncoprotein. *EMBO J.*, **11**, 5013–5020.

MIYASHITA T, HARIGAI M, HANADA M AND REED JC. (1994). Identification of a p53-dependent negative response element in the bcl-2 gene. *Cancer Res.*, **54**, 3131–3135.

MULLINS KB AND FALOONA F. (1987). Specific synthesis of DNA *in vitro* via a polymerase-catalyzed chain reaction. *Methods Enzymol.*, **155**, 335–350.

NAVONE NM, TRONCOSO P, PISTERS LL, GOODROW TL, PALMER JL, NOCHOLS WW, VON ESCHENBACH AC AND CONTI CJ. (1993). p53 protein accumulation and gene mutation in the progression of human prostate carcinoma. *J. Natl Cancer Inst.*, **85**, 1657–1669.

PEEHL DM. (1993). Oncogenes in prostate cancer. *Cancer*, **71**, 1159–1164.

SCHARER E AND IGGO R. (1992). Mammalian p53 can function as a transcription factor in yeast. *Nucleic Acids Res.*, **20**, 1539–1545.

SHARKEY D, SCALICE E, CHRISTRY K, ATWOOD SM AND DAISS JL. (1994). Antibodies as thermolabile switches: high temperature triggering for the polymerase chain reaction. *Biotechnology*, **12**, 506–509.

THOMPSON SJ, MELLON K, CHARLTON RG, MARSH C, ROBINSON M AND NEAL DE. (1992). p53 and Ki-67 immunoreactivity in human prostate cancer and benign hyperplasia. *Br. J. Urol.*, **69**, 609–613.

VAN VELDHUIZEN PJ, SADASIVAN R, GARCIA F, AUSTENFELD MS AND STEPHENS RL. (1993). Mutant p53 expression in prostate carcinoma. *The Prostate*, **22**, 23–30.

VISAKORPI T, KALLIONIEMI OP, HEIKKINEN A, KOIVULA T AND ISOLA J. (1992). Small sub-group of aggressive, highly proliferative prostatic carcinomas defined by p53 accumulation. *J. Natl Cancer Inst.*, **84**, 883–887.

VOGELSTEIN B AND KINZLER KW. (1992). p53 function and dysfunction. *Cell*, **70**, 523–526.

XIONG Y, HANNON GJ, ZHANG H, CASSO D, KOBAYASHI R AND BEACH D. (1993). p21 is a universal inhibitor of cyclin kinases. *Nature*, **366**, 701–711.